# Impact of COVID-19 pandemic on emergency department visits for drowning at one Texas children’s hospital

**DOI:** 10.5249/jivr.v17i1.1920

**Published:** 2025-01

**Authors:** Molly B. Johnson, Diane Bao, Supriyanka Addimulam, Karen Piper, Karla A.Lawson

**Affiliations:** ^*a*^ Trauma and Injury Research Center, Dell Children’s Medical Center, Austin, TX, USA.; ^*b*^ Kinesiology Department, School of Nursing and Health Professions, University of the Incarnate Word, San Antonio, TX, USA.; ^*c*^ School of Public Health Austin, UT Health, The University of Texas Health Science Center at Houston, Austin, TX, USA.; ^*d*^ Department of Surgery and Perioperative Care, Dell Medical School, University of Texas at Austin, Austin, TX, USA.

**Keywords:** Drowning Submersion, Injury prevention, Emergency department

## Abstract

**Background:**

Drowning is a leading cause of death for children. Fatal and nonfatal drowning incidents often require emergency care. During the COVID-19 pandemic, there were many changes to people’s daily activities due to restrictions on public places, such as swimming pools, and to personal precautions taken to avoid exposure to COVID. This study aimed to assess differences in emergency department (ED) visits and patient demographics, scene factors, and drowning severity for children treated for drowning during the COVID pandemic compared with the two prior years at one pediatric hospital in Texas.

**Methods:**

This 4-year retrospective study used data from a hospital-maintained submersion registry of patients treated for drowning to assess differences in drowning ED visits and patient factors during COVID (April 1, 2020 - March 30, 2022) and the two years immediately prior to the COVID pandemic (April 1, 2018 - March 30, 2020).

**Results:**

Of 166 patients treated for drowning, 85 were pre-COVID and 81 were during COVID. Results showed a decrease in total ED visits, but no change in drowning ED visits or the rate of drowning visits per 10,000 ED visits. During the pandemic, children treated for drowning were more likely to have private insurance and less likely to be uninsured. There were no significant differences in other patient or incident factors.

**Conclusions:**

Results suggest patients continued to seek emergency treatment for drowning during the pandemic despite decreases in overall ED usage. Further studies are needed to explore potential shifts in the patient population or the setting where drownings occurred.

## Introduction

According to the Centers for Disease Control and Prevention (CDC), drowning is the leading cause of death among children aged 1-4 in the United States (USA) and the second most common cause of death by unintentional injury among children aged 5-14.^1^ Even nonfatal pediatric drowning events may require emergency care or hospitalization and ultimately result in neurological damage.^2^ Children with drowning-related morbidity may suffer from long-term issues with behavior, communication, executive function and learning.^3^

The CDC highlights several risk factors for pediatric drowning, such as not being able to swim, having a pool without effective fencing, or lacking proper supervision.^1^ Long-term injury prevention initiatives may help to address some risk factors. Local and global events may also impact drowning risk. For instance, there is a need to better understand how increases in flooding and extreme weather associated with climate change may change drowning risk.^4^ The coronavirus disease of 2019 (COVID-19) pandemic may have also changed overall drowning rates or shifted who was at-risk. 

The COVID-19 pandemic drastically altered social activities and everyday behaviors.^5^ For instance, to prevent the spread of COVID-19, many indoor and outdoor community swimming pools in Texas closed in March 2020.^6^ During the COVID-19 pandemic, caregivers of toddlers in the USA reported delaying swimming lessons, swimming less often with their toddler, and swimming in different locations than they did before the pandemic.^7^ The COVID-19 pandemic also drastically influenced parents’ travel and leisure decisions, directly impacting children’s access to pools and bodies of water.^8^ Lower levels of international and regional travel coupled with decreased access to community swimming pools may have led to a reduction in drownings during the COVID-19 pandemic compared to the years directly previous. On the other hand, the unaffordability and closure of swimming lessons may have deprived children of valuable opportunities to practice swimming, potentially increasing the risk of drowning. 

The aim of this study was to explore whether there were changes in the number of children treated for drowning at one pediatric hospital in Texas during the two years during the COVID pandemic compared to the two-year period pre-COVID. Additionally, we explored whether there were differences in the characteristics of the patients, the incident, or the injury severity between the two time periods.

## Methods 

This retrospective study analyzed data for patients under 18 years of age treated for submersion injury at an urban pediatric hospital with a Level 1 trauma center in Texas, USA. over a four-year time span that included two years during the COVID pandemic (COVID: April 1, 2020 and March 30, 2022) and the two years immediately prior to the COVID pandemic (pre-COVID: April 1, 2018 and March 30, 2020). Patients included in this analysis were those in a registry of submersion injury patients maintained at the hospital for submission to a state-mandated submersion registry. Patients meeting inclusion for this registry were identified by querying hospital administrative databases for patient encounters. Hospital administrative databases were searched for at least one of the following ICD-10 codes: initial encounters (codes ending in A) and having a "1" in the sixth digit indicating "causing drowning and submersion" in the range W16.011-W16.331 and W16.511-W16.831 or having initial encounter codes beginning with W16.41, W16.91, W22.041, W65-W74, X71, X92, Y21, or T75.1. An additional string search was performed on another administrative database variable representing the chief complaint of hospital presentation using the "%drown%" string pattern. Manual retrospective chart review was conducted on identified encounters to ensure inclusion was met. Two authors (MBJ, KAL) abstracted data from the medical records of all Submersion Registry cases, reconciling differences through discussions, and documenting decisions to maintain consistency for all cases. The registry of submersion injury patients is managed using REDCap (Research Electronic Data Capture) electronic data capture tools hosted at Seton Analytics & Health Economics.^9,10^ Ethics approval for the research was granted by the University of Texas at Austin Health Sciences Institutional Review Board (STUDY00001247).

**Data Analysis**

We compared the total number of patients treated for drowning injury, the total number of emergency department (ED) visits as well as the rate of drowning visits per 10,000 ED visits during COVID to pre-COVID. Demographic and drowning scenario data included age, sex, race, ethnicity, insurance status (public insurance category included Medicaid, Medicaid managed care, institutional, and Medical Access Program insurance; private insurance category included any insurance purchased through an employer or privately), setting, whether the water time was planned, whether the child had a pulse when they were removed from the water, whether the child received CPR when removed from the water (e.g. rescue breaths, chest compressions, or pats on the back), whether they were admitted to the intensive care unit (ICU), and drowning outcome. To assess differences between patients during COVID compared with pre-COVID, data on patient demographics, drowning incident scenario factors, and injury severity were analyzed using chi-square and Fisher’s exact test. Data were analyzed using STATA 17. Unknown or missing data were not included in analyses. Unknown or missing data accounted for 2% or less for race, setting, and CPR; 7% for water time planned; 10% for ethnicity; and 32% for pulse.

## Results

**Drowning Visits and Rate**

There were 166 pediatric patients who visited the ED for drowning injury out of 234,983 total pediatric ED patient visits during the four years between April 1, 2018 and March 30, 2022 (Table 1). Of the patients treated for drowning, 51% of the visits were during the two years pre-COVID and 49% of the visits were during COVID. When reviewing all ED visits for any reason, 59% of the visits were pre-COVID and 41% of the visits were during COVID. The number of drowning visits per 10,000 ED visits was 6.13 pre-COVID and 8.40 during COVID. Overlapping confidence intervals for all years indicate no significant differences in drowning visit rates between any of the four years or between two-year COVID and pre-COVID time periods.

**Table 1:** Emergency department visits

	Pre-COVID			COVID			All Years
	Year 1	Year 2	Total	Year 1	Year 2	Total	
**Drowning Visits (n)**	35	50	85	33	48	81	166
**Drowning Visits (%)**	21.1	30.1	51.2	19.9	28.9	48.8	100
**ED Visits**	68,560	70,002	138,562	34,401	62,020	96,421	234,983
**ED Visits (%)**	29.2	29.8	59.0	14.6	26.4	41.0	100
**Rate per 10,000**	5.11	7.14	6.13	9.59	7.74	8.40	7.06
**CI Lower Bound**	3.56	5.30	4.90	6.60	5.71	6.67	6.03
**CI Upper Bound**	7.10	9.42	7.59	13.47	10.26	10.44	8.22

Note: Pre-COVID: April 2018 - March 2020, COVID: April 2020 - March 2022. Rate = number of drowning visits per 10,000 Emergency Department (ED) visits. CI = 95% confidence interval.

**Timeline of Drowning Visits**

**Figure 1:**
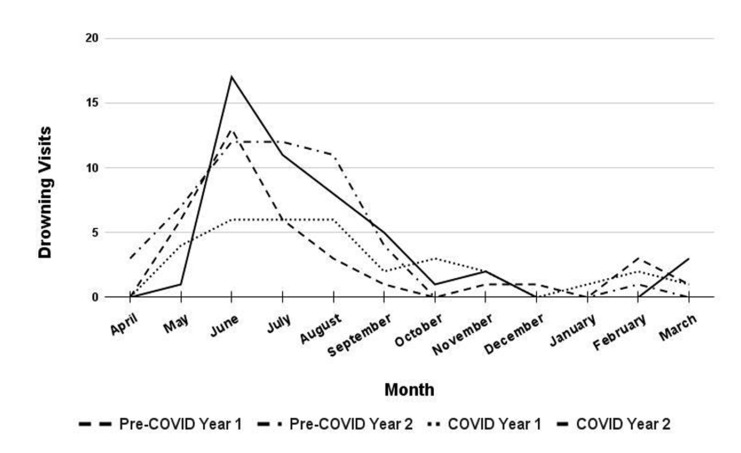
Emergency department drowning visits per month pre-COVID and during COVID

Although drowning numbers and rates did not vary between years, the timeline of when the drownings occurred shifted somewhat during the pandemic compared to prior years (Figure 1). During the first year of COVID, the number of drowning visits in May and June remained lower than during pre-COVID years. During the second summer of COVID, June cases spiked to their highest point of all four years, with a similar month-to-month pattern to pre-COVID years.

**Patient Demographics**

**Figure 2:**
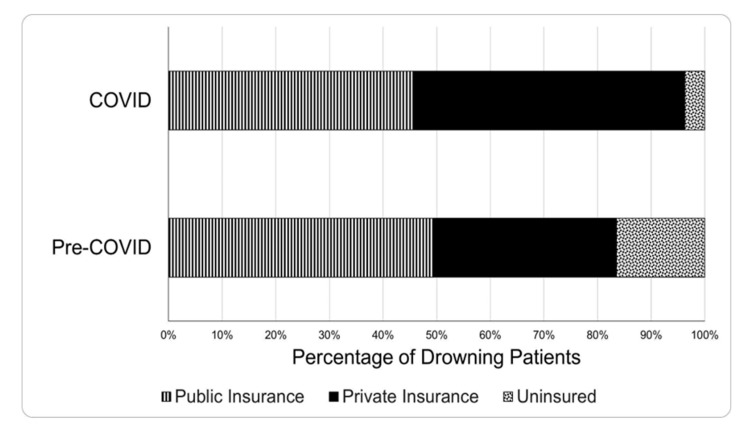
Insurance status of emergency department patients treated for drowning pre-COVID and during COVID

Of patients treated for drowning during the four years studied, 62% were toddlers (1-4 years old), 57% were male, 79% were White, and 41% were Hispanic. There were no significant differences in the demographic characteristics of drowning patients during COVID compared to pre-COVID (Table 2). The insurance status of patients was significantly different during COVID than pre-COVID (Figure 2). Although the percentage of patients on public insurance, such as Medicaid, remained similar during the two time periods, during COVID, patients were more likely to be privately insured and less likely to be uninsured compared to pre-COVID (Table 2).

**Table 2:** Demographic characteristics of pediatric drowning patients

Variable	Description	Pre-COVID		COVID		Total		p-value
		n	%	n	%	n	%	
**Total Drowning Visits**		85	51.2	81	48.8	166	100	-
**Age**	<1 yr	10	11.7	7	8.6	17	10.2	905
	1-4 yrs	52	61.2	51	63.0	103	62.0	
	5-13 yrs	18	21.2	17	21.0	35	21.1	
	14-18 yrs	5	5.9	6	6.6	11	6.6	
**Sex**	Female	35	41.2	36	42.8	71	42.8	.671
	Male	50	58.8	45	57.2	95	57.2	
**Race**	Asian	3	3.5	3	3.8	6	3.7	.868
	Black	9	10.6	11	13.9	20	12.2	
	White	69	81.2	60	76.0	129	78.7	
	Other	4	4.7	5	6.3	9	5.5	
**Ethnicity**	Hispanic	35	44.3	26	37.1	61	40.9	.375
	Not Hispanic	44	55.7	44	62.9	88	59.1	
**Insurance Status**	Uninsured	14	16.5	3	3.7	17	10.2	.008*
	Public Insurance	42	49.4	37	45.7	79	47.6	
	Private Insurance	29	34.1	41	50.6	70	42.2	
**Setting**	Natural Water	8	9.4	13	16.3	21	12.7	.301
	Pool/Spa	67	78.8	55	68.8	122	73.9	
	Bathtub	10	11.8	12	15.0	22	13.3	
**Water Time Planned**	Yes	61	77.2	59	78.7	120	77.9	.828
	No	18	22.8	16	21.3	34	22.1	
**Pulse **	Yes	49	81.7	41	77.4	90	79.7	.570
	No	11	18.3	12	22.6	23	20.4	
**CPR**	Yes	46	54.1	45	57.7	91	55.8	.646
	No	39	45.9	33	42.3	72	44.2	
**ICU Admission**	Yes	16	18.8	13	16.1	29	17.5	.638
	No	69	81.2	68	84.0	137	82.5	
**Outcome**	No Morbidity	75	88.2	71	87.7	146	88.0	.673*
	Morbidity	3	3.5	5	6.2	8	4.8	
	Death	7	8.2	5	6.2	12	7.2	

Notes: For all variables, data that were not documented in medical records or were unknown were not included in analyses. * indicates Fisher’s exact test was used instead of chi-square. ICU = intensive care unit. CPR = cardiopulmonary resuscitation. Pre-COVID timeframe: April 2018 - March 2020, COVID timeframe: April 2020 - March 2022.

**Drowning Context**

During the four years studied, 74% of drownings occurred in pools or spas; 13% occurred in natural water, such as lakes, ponds, or oceans; and 13% occurred in bathtubs. In 78% of cases, the child’s access to water had been planned. There were no significant differences in the drowning setting or planning during COVID compared to pre-COVID (Table 2).

**Severity and Outcome of Drowning Injury**

Of patients treated for drowning during the four years studied, 56% received a CPR-type intervention immediately after being removed from the water and 20% did not have a pulse detected when they were retrieved from the water. After being monitored and treated in the ED, 39% were discharged home, 46% were admitted for less than 24 hrs, and 15% were admitted for more than 24 hrs or expired. There was no morbidity reported for 88% of patients, while 5% had morbidity and 7% experienced a fatal drowning. There were no differences in the severity or outcome of drowning cases during COVID compared to pre-COVID (Table 2). 

## Discussion

This study showed that the total number of ED visits dropped during the COVID pandemic (April 2020 - March 2022) compared to the two pre-pandemic years (April 2018 - March 2020), but that visits for pediatric drowning and the rate of pediatric drowning visits per 10,000 ED visits remained somewhat consistent between time periods. Our results indicate that we have to be cautious about relying on ED visit rates to understand drowning. We saw slightly increased rates during COVID, but they were not attributable to increased drowning visits, but to decreased total ED visits. During the pandemic, children treated for drowning were more likely to be privately insured and less likely to be uninsured compared to children treated for drowning pre-COVID. There were no other differences in demographic characteristics of patients treated for drowning between the two time periods and no differences in incident details or injury severity. 

Our finding that ED visits dropped substantially during COVID compared to pre-COVID is supported by national data showing a drop in ED visits across the USA during the early part of the pandemic, particularly for children.^11^ The relatively consistent drowning visits despite lower overall ED visits during the pandemic suggest that people’s willingness to pursue ED care for drowning incidents did not change. 

In our patient population, we saw a larger proportion of people with private insurance and a lower proportion of people who were uninsured. It is unclear what drove these differences. This could be due to people without insurance avoiding the ED during COVID. Further research is needed to understand insurance shifts in the USA as a whole and in the patient population.

During the pandemic, with many indoor recreational activities restricted, some people may have greatly increased their time spent around home pools or natural water, increasing the exposure and drowning risk of this group.^12^ Prior research on drowning rates for all ages in a series of natural water lakes in the USA showed this trend, with an initial decline in drownings followed by an increase.^13^ Pool and natural water area restriction and re-opening timing could have facilitated a similar trend in Texas.^6^ Our data on pediatric drowning show a lower summer peak the first year of the pandemic and a higher summer peak the second year of the pandemic compared to the two prior years (Figure 1). However, we found that total drowning numbers and rates were not significantly different between time periods and between the first and second years of the pandemic.

Potential changes in where people were swimming might have impacted where people drowned during the pandemic compared to prior years. Changes in family swimming locations were reported during the pandemic.^7^ National data on drowning fatalities for children and adults 29 years old and younger show a 26% increase in natural water drownings in 2020 compared with prior years, but this increase may be more attributed to an increase in drownings in 2020 for those 20-29 years old that was not seen for toddlers.^14^ We saw a higher percentage of drownings in natural water and a lower percentage in swimming pools during the pandemic compared with prior years, but the results did not reach the level of significance (Table 2). Larger datasets are needed to understand potential shifts in drowning settings during the pandemic for all age groups.

It is also important to remain aware of racial disparities in drowning. Black Texans have a higher drowning fatality rate than White Texans for both adults and children under 18 years old.^15^ Across the USA, there are higher drowning fatality rates for Black and American Indian/Alaska Native persons than White persons.^16^ Nationally, racial disparities in drowning widened in 2020 compared to prior years.^14^ Our data represents a small portion of the pediatric population in Texas. The proportion of drowning cases that were Black rose during the pandemic, but this was not significant. Our lack of significant findings could be related to smaller drowning disparities in some age groups of children. In national data for 0-29 year olds, the only age group without disparities in fatal drownings between Black and White persons was 1-4 year olds.^14^ Over 60% of the patients treated for drowning in our data were 1-4 years old, so it is unclear whether our sample size was too small to see racial differences or whether the pandemic impacted race and age in an interactive way.

**Limitations**

There were several factors that could have impacted our results. In the investigation of associations between patient and incident characteristics with time period, some variables (ethnicity, water time planned, pulse) had a higher level of missing data. In addition, this was a single site investigation and power to investigate associations was limited. Additionally, the results may not be transferable to the USA population as a whole or to other regions. Future research could combine data from multiple institutions to increase the statistical power of analyses to further explore the impact of the pandemic on drowning. Another limitation of this study is that many of the patients evaluated for drowning in our dataset had a pulse when removed from the water, did not need CPR, and were discharged from the ED without the need for extensive treatment. It is likely that some of these patients were brought to the ED for evaluation only and did not get water in their lungs or have any respiratory impairment. These patients would not meet the World Health Organization definition of drowning, but might instead be considered rescues.^17^ Further exploration is needed to establish procedures to classify hospital patients as fatal or nonfatal drowning and identify those evaluated for drowning, but who do not have respiratory symptoms.

## Conclusion

This study found no change in the number of children treated for drowning or the rate per 10,000 ED visits in a large urban children’s hospital in spite of large reductions in total ED visits during the pandemic compared with prior years. Children treated for drowning during COVID were less likely to be privately insured and more likely to be uninsured than pre-COVID. There were no significant differences in other demographic, incident, or severity variables.

**Authors Contributions:**Authors MJ, KP, and KL contributed to the study design. All authors contributed to the data analysis and interpretation and to manuscript revision. MJ led manuscript writing. All authors approved the manuscript as submitted.
